# Attention deficit hyperactivity disorder (ADHD) in phenotypically similar neurogenetic conditions: Turner syndrome and the RASopathies

**DOI:** 10.1186/s11689-017-9205-x

**Published:** 2017-07-10

**Authors:** Tamar Green, Paige E. Naylor, William Davies

**Affiliations:** 1 Center for Interdisciplinary Brain Sciences Research, Stanford University School of Medicine, Stanford, USA; 2 Department of Clinical Psychology, Palo Alto University, Palo Alto, CA USA; 3 Medical Research Council Centre for Neuropsychiatric Genetics and Genomics and Division of Psychological Medicine and Clinical Neurosciences, School of Medicine, Cardiff University, Cardiff, UK; 4 School of Psychology, Cardiff University, Tower Building, 70, Park Place, Cardiff, CF10 3AT UK; 5 Neuroscience and Mental Health Research Institute, Cardiff University, Cardiff, UK

**Keywords:** Attention deficit hyperactivity disorder, Neurofibromatosis type 1, Noonan syndrome, RASopathies, Turner syndrome, X chromosome

## Abstract

**Background:**

ADHD (attention deficit hyperactivity disorder) is a common neurodevelopmental disorder. There has been extensive clinical and basic research in the field of ADHD over the past 20 years, but the mechanisms underlying ADHD risk are multifactorial, complex and heterogeneous and, as yet, are poorly defined. In this review, we argue that one approach to address this challenge is to study well-defined disorders to provide insights into potential biological pathways that may be involved in idiopathic ADHD.

**Main body:**

To address this premise, we selected two neurogenetic conditions that are associated with significantly increased ADHD risk: Turner syndrome and the RASopathies (of which Noonan syndrome and neurofibromatosis type 1 are the best-defined with regard to ADHD-related phenotypes). These syndromes were chosen for two main reasons: first, because intellectual functioning is relatively preserved, and second, because they are strikingly phenotypically similar but are etiologically distinct. We review the cognitive, behavioural, neural and cellular phenotypes associated with these conditions and examine their relevance as a model for idiopathic ADHD.

**Conclusion:**

We conclude by discussing current and future opportunities in the clinical and basic research of these conditions, which, in turn, may shed light upon the biological pathways underlying idiopathic ADHD.

## Background

ADHD (attention deficit hyperactivity disorder, also known as hyperkinetic disorder) is currently defined in diagnostic manuals such as DSM-5 [1] and ICD-10 [2] by a collection of persistent and impairing cognitive and behavioural symptoms, notably inattention, hyperactivity and pathological impulsivity. ADHD is the most common neurodevelopmental disorder in the USA, affecting up to 10% of children and exhibiting a pronounced male-biased diagnostic prevalence [3]. The biological and environmental mechanisms underlying ADHD risk are multifactorial, complex and heterogeneous [4, 5] and, as yet, are poorly defined. There is accumulating evidence that ADHD is associated with structural and functional abnormalities across multiple brain circuits, notably reduced volume of the basal ganglia, thinning of the frontal and parietal cortex, and functional connectivity between these regions [6]. In the absence of an established pathophysiology, we are limited to using treatments that target symptoms rather than core cellular and molecular abnormalities; as a result, the efficacy and side-effect profile of these therapies is sub-optimal and treatments cannot directly influence the course of the disorder.

Identifying and characterising the many dysfunctional biological pathways that culminate in the development of ADHD is a difficult, perhaps intractable, problem. On the one hand, studying children and adults with idiopathic ADHD is hindered by limited sample sizes and other issues that stem from within-group heterogeneity. On the other hand, it is difficult to model the complex cognitive and behavioural abnormalities seen in individuals with ADHD in animal models where experimental control is more feasible. The study of well-defined neurogenetic conditions in man circumvents, to some extent, challenges associated with etiological heterogeneity, whilst allowing the measurement of complex human behaviours in children and adult with these conditions.

**Fig. 1:**
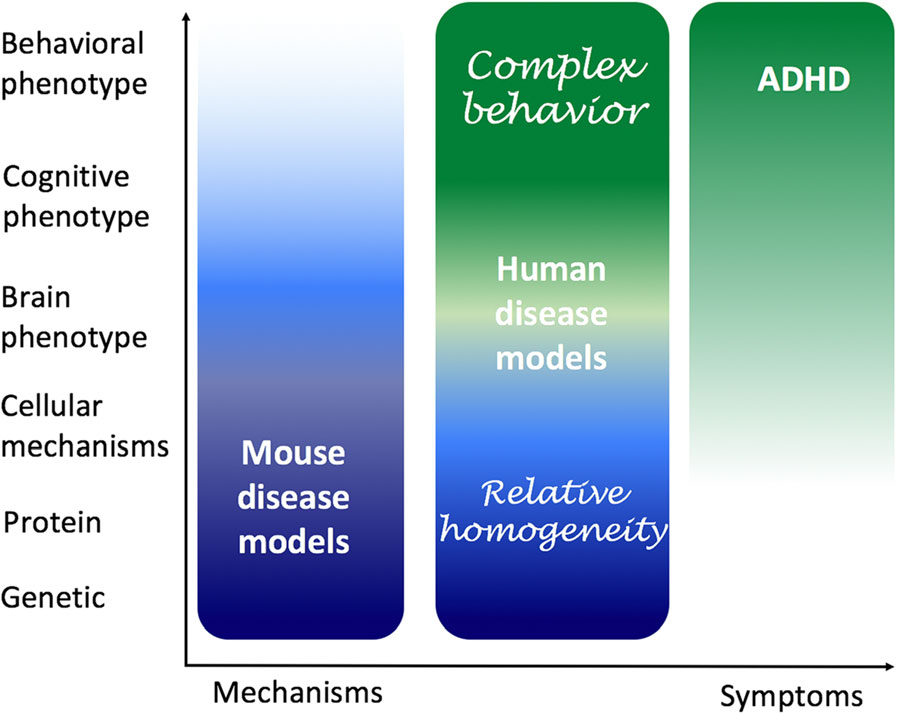
Human disease model as a framework for the study of ADHD. This framework theoretically models complex human behaviour not available through mouse models for relatively genetically homogeneous populations compared to the study of heterogenic populations with ADHD. The structure of the current review is provided on the *Y* axis, including the cognitive, behavioural, neural and cellular phenotypes associated with Turner syndrome, Noonan syndrome, and neurofibromatosis type 1

In this review, we examine the premise that human neurogenetic conditions that are associated with increased ADHD rates may help to provide converging evidence for biological pathways involved in the development of idiopathic ADHD. Specifically, we compare two phenotypically similar classes of human genetic conditions that may serve as such ‘experimental models’: Turner syndrome (TS) and the RASopathies. Of the known RASopathies, we focus on Noonan syndrome (NS) and neurofibromatosis type 1 (NF-1), conditions about which most data has been collated in the literature. Moreover, whilst ADHD symptomatology is reported across all of the RASopathies, including Costello syndrome, LEOPARD syndrome, and cardiofaciocutaneous syndrome (CFC), these disorders are associated with pervasive effects on global cognitive function that might confound more specific genetic effects on ADHD-related symptoms [7]. TS, NF1 and Noonan syndrome are mechanistically distinct conditions which result in phenotypically aberrant behaviours and cognitive processes, but individuals affected by these conditions have similar overall cognitive profiles which lie within the normal range (Table 1). Importantly, the two classes of condition show striking overlap with regard to their associated anatomical/physiological phenotypes (Table 1) and, as such, there may feasibly be some degree of convergence across both with respect to the biological mechanisms underlying ADHD risk; clarifying the level at which the genetically distinct aetiologies in TS and the RASopathies might ultimately impact upon common brain substrates underlying ADHD-related phenotypes (e.g. molecular, cellular or brain circuit) will comprise an important avenue for future research. We start by critically reviewing the existing clinical and animal model literature relating to cognitive, behavioural, neural and cellular phenotypes in these conditions (Fig. 1) and examine their relevance as a model for idiopathic ADHD.

**Table 1:** The genetic mutations, protein products, clinical features, and general intellectual functioning associated with TS, NS and NF1

	Genetic mutation	Protein	Clinical phenotype	General cognitive functioning
Turner syndrome	X chromosome partial (mosaic)/complete deletion	Reduced expression of gene products encoded by X-linked genes escaping X-inactivation [121]	Renal/endocrine problems, cardiac defects, short stature, webbed neck, eyelid ptosis, increased inter-nipple distance [114, 116]	Mosaic/partial X chromosome absence: VIQ: 96.2 ± 15.9 PIQ: 79.5 ± 18.8 Complete X chromosome absence: VIQ: 106.4 ± 14.4 PIQ: 82.1 ± 15.9 [48]
Noonan syndrome	- *PTPN11* (50%) ⇒	⇑ Shp2 tyrosine phosphate enzyme	Short stature, webbed neck, eyelid ptosis, increased inter-nipple distance, cardiac defects, bleeding disorders [114, 115, 117, 118]	VIQ: 82.3 ± 20.0 PIQ: 87.1 ± 23.0 (May vary depending upon specific mutation [119, 120])
	- *SOS1* (10–15%) ⇒	⇑ “Son Of Sevenless 1” protein		
	- *RAF1* (5–10%) ⇒	⇑ Serine-threonine kinase activating MEK1/MEK2		
	- *KRAS* (0–5%) ⇒ [111]	Missense mutation KRAS Isoform B [118]		
	- Other rare mutations			
Neurofibromatosis type 1	*NF1* gene microdeletion (chromosome 17q11) [112]	⇓ Neurofibromin [113]	Café-au-lait spots, intertriginous freckling, Lisch nodules, neurofibromas, optic pathway gliomas, distinctive bony lesions [111-113]	VIQ: 91.9 ± 14.7 PIQ: 91.1 ± 12.8 [11]

*VIQ* verbal IQ, *PIQ* performance IQ)

## ADHD-related behavioural and cognitive phenotypes in TS, NF1 and NS

Significantly increased (~18-fold) rates of ADHD have been reported in TS (~24%) relative to the general female population (1.3%) [8] whilst substantially elevated rates of ADHD have also been reported in several of the RASopathies, including NF1 and NS, relative to the general population [9, 10]. For example, it has been reported that in children with NF1, 38–49% meet diagnostic criteria for ADHD, whilst an even larger proportion experience considerable difficulties with sustained attention [9, 11]. Children with NF1 also exhibit high rates of explicit autism spectrum disorder (~25% of cases) and autistic symptoms (~50% of cases), but these diagnoses appear dissociable from ADHD-related behaviours [12]. ~31% of children with NS have been reported to meet diagnostic criteria for ADHD [10]. It should be acknowledged that there may be an over-estimation of psychological issues in TS, NF1 and NS populations arising from an ascertainment bias whereby individuals with neurogenetic conditions are monitored and assessed disproportionately deeply compared to members of the general population.

The presentation of ADHD-related symptoms can vary between TS, NF1 and NS. Girls with TS and ADHD present with pronounced hyperactivity (comparable to levels seen in idiopathic ADHD in boys) [13] and are more likely to be diagnosed with the hyperactive-impulsive presentation of the disorder [8]. Attention and executive function deficits are, however, also apparent in this population, and appear to be independent of other cognitive deficits characteristic of this population such as visuospatial impairment and general cognitive ability; interestingly, there appears to be a bimodal distribution within the TS group with regard to one measure of attentional function [10]. In NF1 and NS, the primary ADHD-related symptoms appear to be difficulties with attention. In both groups, parental reports indicate that children have clinically significant difficulties with self-monitoring (36.5% children with NF1 and 38% of children with NS) and planning-organisation (42.9% children with NF1 and 38% with NS) [10, 14]. Direct neuropsychological measures of ADHD-related behaviours also indicate that both children with NF1 and NS experience significant difficulties with sustained attention and inhibition. However, obtaining accurate measures of ADHD-related behaviours in the RASopathies is difficult; in some studies, assessments of children with severe ADHD were excluded due to concerns regarding their validity [10], and there is some degree of heterogeneity within the clinical population and in terms of the assessment measures used (and the reporting of associated results).

An important question regarding ADHD-related phenotypes in these syndromes is whether they are the culmination of relatively discrete disruption to molecular, cellular and neural pathways, or whether they are simply a subset of the many adverse neurobehavioural consequences arising from gross brain maldevelopment; the fact that IQ is generally preserved across all three conditions indicates the former. Overall therefore, TS, NF1 and NS might be regarded as behavioural models of specific subtypes, or symptoms, of ADHD in the absence of general cognitive impairments. Currently, there is little clinical and neuropsychological information available that may indicate areas of commonality and difference across the three syndromes, and there is undoubtedly a need for further research in this area. Parallel in vivo and in vitro studies such as neuroimaging and animal/cellular model work may enable us to understand how similarities and differences in neuroanatomy, neurophysiology and neurochemistry across the three syndromes can contribute towards commonalities and dissociations in behavioural and cognitive phenotypes.

## Brain phenotype in TS, NF1 and NS: clinical and animal model evidence

In TS, although total brain volume appears comparable to that of healthy controls, there is evidence for decreased volumes of the cortex (particularly in parietal and occipital regions), hippocampus, thalamus and basal ganglia [15, 16]. The structure of the parietal lobe [16–18] and its connectivity with other cortical regions (notably the frontal cortex) [19, 20] appear to differ between individuals with TS and controls. In addition, task-based [21–23] and resting-state [24] functional connectivity measures between the frontal and parietal cortex are reduced in girls with TS. Together, these data provide a possible neural mechanism underlying attention problems and hyperactivity in TS.

Ongoing studies in mouse models with varying sex chromosomal complements are attempting to refine the brain sites that are most sensitive to X and Y chromosome dosage and that may therefore play a role in sex-biased cognitive constructs, including vulnerability to neurodevelopmental disorders such as autism and ADHD; interestingly, these appear to include systems previously implicated in ADHD vulnerability including the forebrain cholinergic system and cerebelo-pontine-thalamo-cortical circuitry [25]. The X-monosomic 39,XO mouse model [26] appears to recapitulate, to some extent, the parietal cortex and striatal structural abnormalities reported in TS [25]. However, it should be noted that the extrapolation of any findings from this work to humans is limited by the fact that there are far fewer X-escapees in the mouse than in human, and the physiological consequences of X-monosomy in the mouse are likely to be different, and generally less severe, than in human [27].

At a structural level, the brains of subjects haploinsufficient for NF1 appear grossly normal, although hyperintensities, possibly reflecting aberrant myelin formations, are seen in patient MRI scans [28]. In the basal ganglia, thalamus and brainstem of patients, such hyperintensities tend to resolve over time (possibly as a consequence of improved efficiency within white matter tracts) and this resolution appears to be related to cognitive improvements, indicating likely pathogenicity [29]. There is some, albeit limited, evidence for aberrant brain structure and function in NF1, compared to controls. Individuals with NF1 may exhibit increased total grey [30] and white [31] matter volume, larger hippocampal and pallidum volumes bilaterally [32], aberrant white matter microstructure (as indexed by increased diffusivity) [33] and abnormal functional connectivity between regions underlying social and cognitive function [34], particularly in the anterior-posterior axis [35]. Mouse models in which NF1 is conditionally ablated in embryonic cerebellar progenitors or neonatal granule neuron progenitors exhibit abnormal cerebellar layering and structure [36], a finding which indicates a critical role for NF1 in normal cerebellar development, and which suggests a possible neural mechanism for some of the motor problems commonly seen in NF1.

The neurobiology of patients with NS has been studied to a limited extent (often in the context of a predisposition to cancer), and genotype-phenotype correlations have rarely been reported. Brain structure in NS appears grossly normal, but neurological abnormalities have been reported in some cases. Chiari I malformations have been noted in some cases of NS [37], whilst a boy with NS and a mutation in RAF1 was reported to exhibit external hydrocephalus and abnormal cerebrovascular pathology [38]. An adult female harbouring a *KRAS* mutation followed over the course of several years displayed epilepsy, and hippocampal sclerosis and atrophy [39]; neuroimaging and animal model work (see below) demonstrates that hippocampal pathology appears to be a feature common to NF1 and other RASopathies. Individuals with NS and a newly reported mutation in *SHOC2* exhibit a relative megalencephaly, enlarged subarachnoid spaces, a relatively small posterior fossa, and a high rate of cerebellar tonsillar ectopia [40].

## Candidate molecular mechanisms underlying increased rates of ADHD in TS

Neuropsychological differences between neurotypical 46,XX females and females with TS could potentially arise due to one or more fundamental genetic mechanisms.

First, a plausible reason for increased ADHD rates in TS stems from the fact that these individuals, like males, are functionally hemizygous for some, or all, X-linked genes. Hence, the effects of X-linked mutations or polymorphisms that elicit ADHD-related phenotypes will be apparent, and will not be moderated by the effects of the second X-linked allele. Candidate gene studies have previously implicated polymorphisms within the X-linked *MAOA* and *HTR2C* genes in phenotypes associated with ADHD [41, 42]; recent recognition that the X chromosome must also be incorporated in genomewide association studies together with the development of statistical tools to perform such analyses (e.g. XWAS [43]), is likely to drive the generation unbiased evidence for association between multiple X-linked genetic variants and ADHD-related phenotypes in the near future.

The second genetic mechanism that may explain why ADHD rates are higher in TS than healthy control subjects is X-linked gene dosage. The epigenetically mediated process of X-inactivation silences one of the two X chromosomes within a female cell to ensure dosage equivalence (and hence gross phenotypic equivalence) to male cells where only one X chromosome is active [44]. However, this silencing process is not complete, and ~15–20% of human X-linked genes are thought to escape X-inactivation to some extent, including both in the pseudoautosomal regions and the X-specific portion of the chromosome [45]; for this subset of genes, expression levels will be higher (though not necessarily double) in female than male cells due to the fact that expression proceeds from two X-linked alleles in females, but only one in males. If reduced dosage for one or more of these so-called ‘X-escapees’ confers ADHD risk, then we might expect individuals with just one X-linked allele (i.e. males and females with TS) to exhibit greater vulnerability than 46,XX females. Work in animal models has begun to identify X-escapees that might influence increased ADHD risk in TS via gene dosage mechanisms (see below). For TS subjects possessing one intact X chromosome and a second X chromosome with a terminal or interstitial chromosomal deletion, the second X chromosome may be preferentially inactivated [46]; this process may both skew the relative expression of X-linked alleles from the paternally and maternally inherited X chromosomes, and subtly affect the expression of X-escapees.

The final genetic mechanism that may explain the elevated ADHD rates in TS is the parental origin of the X chromosome [47]. Briefly, there is limited, somewhat contentious (e.g. [48]), evidence from human [46] and mouse [49] studies that possessing a single maternally inherited X chromosome (45,X^M^) results in poorer social function, and a greater degree of behavioural inflexibility, than inheriting a single paternally inherited X chromosome (45,X^P^). Neurotypical 46, XX females inherit one X chromosome from either parent, and therefore any deleterious effects of the maternally inherited X chromosome may be compensated for by the presence of the paternally inherited X chromosome. Impaired social function, aberrant behavioural inhibition and behavioural inflexibility is commonly seen in individuals with ADHD [50]. As a large proportion (~70%) of X-monosomic women inherit their single X chromosome maternally, we might expect the TS group as a whole to be more severely affected with regard to these ADHD-related brain and behavioural measures than 46,XX females. This ‘parent-of-origin’ idea is not supported by data from the one, rather small, published study that has explicitly tested whether rates of ADHD diagnoses in individuals with 45,X^P^ and X^M^ karyotypes differ (although behavioural flexibility was not assayed in any depth) [8]; however, there is some evidence from TS that parent-of-origin effects may influence aspects of cognition perturbed in ADHD [48, 51].

The genetic mechanisms above could potentially influence brain function directly. Alternatively, they could exert effects on neurodevelopment and brain function indirectly through affecting the development and function of other organs. TS is commonly associated with impaired ovarian formation (and hence reduced levels of systemic reproductive hormones); the condition is also linked to abnormalities in levels of circulating growth hormone and thyroid hormones [52]. An important question is whether the ADHD-related symptoms and cognitive deficits in TS, are influenced primarily by direct genetic effects, primarily by indirect, hormonally mediated effects, or by an equal contribution of the two pathways. There are a number of lines of evidence which suggest that the first option may be the most plausible. For example, many TS cognitive deficits are maintained throughout development despite considerable fluctuations in levels of circulating hormones across the lifecourse; moreover, cognitive deficits are not alleviated to any significant extent by the administration of oestrogens, androgens or growth hormone (e.g. Freriks et al., 2015 [53]), and women with premature ovarian failure who exhibit similar hormonal profiles to women with TS do not present with a similar constellation of cognitive deficits [54]. However, some modulatory role for hormonal (and environmental) influences on cognition, particularly during foetal development or early childhood, is likely.

Given the data above, which sex-linked genes may be regarded as candidates for influencing attentional phenotypes in TS, and which neural circuitry may they influence? Y-linked genes that may be of particular interest with respect to ADHD include the male-determining gene *SRY* (which is expressed in dopaminergic neuron-rich brain regions and is duplicated in cases of ADHD), and *NLGN4Y* and *PCDH11Y*, which encode cell membrane molecules important in maintaining synapse integrity and cell-cell interactions respectively, and both of which have been implicated as candidate genes for neurodevelopmental disorders [55]. In TS these mechanisms might come into play in individuals where mosaic Y-linked sequences inherited from their father are present.

Deletion mapping strategies (whereby the phenotypes of TS subjects possessing a variety of partial deletions of the second X chromosome are assessed) have been useful for identifying genomic regions that may potentially underlie X-linked gene dosage effects on executive function and social cognition. Zinn and colleagues have shown that individuals in which a small portion of the short of the X chromosome was missing (Xp22.3) consistently demonstrate abnormal neurocognitive function, including across an assay of attention (TOVA) on which subjects with ADHD are impaired [56]. Of the 31 genes within this interval, the authors suggested *STS* and *NLGN4X* (the X-linked homologue of *NLGN4X*) as candidate genes. *STS* escapes X-inactivation in both humans and mouse [57] and consequently is more highly expressed in foetal and adult female than male brain (unpublished results). Recent cross-species work has strengthened the evidence for this gene as a mediator of attention. Specifically, X-monosomic mice (which exhibit neither short stature nor gross hormonal abnormalities) exhibit attentional deficits that can be rescued by the presence of a small chromosome housing *Sts* [58]; moreover, mice lacking the *Sts* gene, or mice in which the associated steroid sulfatase enzyme is inhibited, show attentional deficits and other phenotypes of relevance to ADHD [42, 47, 59, 60]. In a demonstration of the power of studying rare neurogenetic conditions to understand the pathophysiology underlying more common idiopathic disorders, we, and others, have shown, on the basis of work predicting steroid sulfatase involvement in (in)attention from TS and related mouse models, that polymorphisms within *STS* are associated with the inattentive symptom count in boys diagnosed with idiopathic ADHD [61, 62].

Deletion mapping in TS has also revealed a small dosage-sensitive locus at Xp11.3 that is linked to impaired emotion recognition and increased orbitofrontal cortex grey matter and amygdala size [63]; follow-up genetic association analyses have implicated a variant within the *EFHC2* gene in these phenotypes in both females with TS and healthy males [64, 65], although the data from TS is somewhat controversial [66]. There is some evidence that individuals with ADHD exhibit abnormal orbitofrontal cortex grey matter volumes, and related deficits in emotion perception [67–70]; testing for genetic association within *EFHC2* and these phenotypes within idiopathic ADHD therefore represents a sensible future avenue for research. To date, no X-linked genes whose expression is significantly influenced in a parent-of-origin dependent manner have been identified in human somatic tissues, including brain. Interestingly, however, the closest human orthologue of the genomically imprinted maternally expressed mouse gene *Xlr3b* identified in a mouse model of TS [49], *FAM9B*, is located at Xp22.3 adjacent to the interval for TS neurocognitive function indicated by deletion mapping. Whilst *FAM9B* is apparently not expressed in the brain, it is expressed in other reproductive tissues [71] and is associated with levels of serum androgens in men [72]. Investigating whether *FAM9B* activity may partially explain the X-linked POE on behaviour seen in TS, and assessing whether this gene may play a role in ADHD-relevant phenotypes related to circulating androgens [41] will also be a worthwhile avenue for further studies.

The bimodal peak in attention problems within a sample of participants with TS reported recently [13] is intriguing and could be explained in a number of ways. First, the distinct sub-groups may have differed subtly in their demographic characteristics (e.g. age, or socioeconomic group) or with respect to their medication regimes, although comparing the two subgroups on these measures did not find significant differences (*p >* 0.5). Alternatively, the two groups may have differed in this cognitive domain as a function of their (epi)genotype. Theoretically, the bimodal distribution might have arisen due to phenotypic differences between 45,X^P^ and 45,X^M^ subjects, although, overall, and consistent with previous data [8], the authors found no evidence for parent-of-origin of the X chromosome influencing ADHD rates within the TS sample. More plausibly, this particular pattern of data may be most parsimoniously explained by hemizygosity, with subjects possessing one or other alleles of X-linked genes affecting attentional processes. Given the converging evidence from human and mouse studies for an attentional role for *STS* (and inherent genetic polymorphisms) described above, we hypothesise that the two groups may be distinguished by their genotype at this locus, or by systematic levels of metabolites dependent upon enzyme function (e.g. dehydroepiandrosterone, DHEA).

An alternative method to deletion mapping for identifying candidate genes underlying ADHD-related phenotypes in TS is to compare gene expression in case samples with that of neurotypical controls. Comparing tissue expression between the two groups is difficult for a number of reasons including the unavailability of the most pertinent tissue (i.e. brain), and obtaining TS samples from donors with consistent ages, karyotypes, hormonal profiles, and treatment histories. A comparison of cell-free RNA levels from the amniotic fluid surrounding TS or euploid female foetuses, which circumvents some of the aforementioned problems, has hinted at dysregulation of the hematologic/immune system in TS and has highlighted a number of specific X-linked and autosomal candidate genes for TS phenotypes [73]; of these, three have previously been tentatively linked to idiopathic ADHD by genetic association studies (*FEN1/ELOVL6* [74], *GSK3B* [75] and *BAIAP2* [76]) and as such may be regarded as potential modulators of ADHD risk both within TS and within the general population. A comparison of gene expression in induced pluripotent stem (iPS) cells derived from individuals with TS or 46,XX individuals has revealed that X-monosomy results in extensive effects on the whole transcriptome, with most impact upon genes involved in central nervous system development [77]; of the most highly differentially expressed genes (>20-fold change), the two with the most relevance to ADHD are *DPP6* [78] and *MYT1L* [79]. Clearly, gene expression differences in iPS cells may not provide an accurate representation of gene expression differences that occur in the developing and mature brains of TS and 46,XX individuals, and, as such, future studies might aim to compare the expression of differentiated neural cells with a view to identifying more plausible candidates for TS cognitive phenotypes.

## Candidate cellular mechanisms underlying increased rates of ADHD in RASopathies

Several converging lines of evidence have implicated GABAergic dysfunction in NF1 cognitive pathology. A seminal series of studies focussing on the hippocampus showed that mice heterozygous for a null *NF1* mutation exhibited excessive Ras activity, enhanced ERK and synapsin I phosphorylation and increased GABA-mediated inhibition, and consequently specific deficits in long term potentiation of potential relevance to learning deficits [80, 81]; the aberrant phenotypes could be partially rescued by administration of a GABA antagonist. More recent work has shown that GABA-mediated inhibition in NF1-deficient mice may influence working memory performance via effects on frontostriatal circuitry, a result consistent with the observation that NF1 patients exhibit hypoactivation of this system [82].

Of particular relevance to this review, NF1-deficient mice exhibit attentional impairments in a stimulus detection task dependent upon prefrontal cortex function [83]. The extent to which the attentional deficits seen in these mice are a result of GABAergic dysfunction remains to be formally tested e.g. via pharmacological manipulations. Consistent with the notion of a brain region-specific GABAergic deficit influencing ADHD-related phenotypes in NF1, work in humans has shown that NF1 patients exhibit impaired impulse control, alterations in electroencephalographic (EEG) signatures of basic sensory and visual processing [84], and reduced GABA (but not glutamate) levels in the medial frontal and occipital cortex [85]; only in the medial frontal cortex were GABA levels correlated with impulse control, and in the opposite direction in NF1 patients and controls. Impairments in social cognitive function are frequently seen in ADHD cases, as discussed above. Work in heterozygous NF1 knockout mice in which the MAP kinase pathway was over-activated in neurons of the amygdala and frontal cortex, showed a selective social learning deficit and disruption to GABA and glutamatergic neurotransmission [86]; this study further implicates effects on excitatory and inhibitory neurotransmitter systems as being key to ADHD-related phenotypes in NF1.

Work in animal and cellular models have also shown that disruption of the Ras signalling cascade has downstream effects on dopaminergic function that may partially explain the cognitive deficits associated with NF1. Most germane to the ‘attentional’ focus of this review, is the observation that NF1-deficient mice exhibit a deficit in non-selective and selective attentional function in the absence of hyperactivity [87], a phenotype that is associated with reduced striatal dopamine levels; both the behavioural and neurochemical phenotypes could be ameliorated by the administration of methylphenidate, a dopamine reuptake inhibitor used therapeutically in ADHD cases. Consistent with this, NF1-deficient mice perform poorly on spatial and learning tasks that are dependent upon dopaminergic function, and neural progenitor cells from NF1 patients exhibit reduced dopamine levels [88]. In terms of molecular pathways, the altered gene expression profile exhibited in the hippocampus of heterozygous NF1 knockout mice has implicated aberrant interactions between neurofibromin, the amyloid precursor protein and the dopamine receptor Drd3 as being important in NF1 psychopathology [89, 90]. Finally, a recent study has shown that pan-neuronal knockdown of *NF1* in Drosophila is associated with locomotor hyperactivity (an ADHD-like phenotype) that could be ameliorated by the administration of methylphenidate [91].

Brain and behavioural phenotypes in NF1 patients may also be influenced by altered levels of circulating glucocorticoids. It has recently been shown that deletion of the NF1 gene in the adrenal cortex increases circulating levels of stress hormones (corticosterone/cortisol) in mouse models and patients [92]. In individuals with ADHD alone, basal cortisol levels tend to be equivalent to those seen in non-affected individuals [93] and therefore increased basal stress hormone levels are unlikely to explain the increased propensity to ADHD in NF1. Existing research on the genetics, cellular biology and animal models of NS provide compelling evidence regarding *PTPN11* effects in the central nervous system. At the cellular level, these studies show that Shp2, encoded by the PTPN11 gene, reduces myelination of axons [94] and is associated with deficits in long-term potentiation (LTP) and increased excitatory synaptic function in hippocampal neurons [95]. In the mouse brain, altered Shp2 expression results in subtle increases in neuron cell density and number, and decreases in astrocyte cell density and number in the hippocampus and forebrain [96]. Behaviour in the Shp2-deficient mouse model of NS has been described as inattentive/hyperactive [95, 97], with cognitive deficits in memory and learning [95].

## A common pathophysiology for ADHD risk in TS and the RASopathies?

Given the overlap in clinical and neuropsychological features between Turner syndrome and the RASopathies (notably with regard to attention deficits/ADHD risk, spatial deficits, motor problems and dyscalculia), is there any evidence for a shared underlying pathophysiology? There are some emerging data from clinical and model studies suggesting that the phenotypes of NF1-deficient individuals may be dependent upon gender, e.g. female patients are more likely to require treatment for visual decline than male patients, whilst only male NF1 knockout mice appear to show spatial learning/memory deficits, increased Ras activity and dopaminergic abnormalities [98]. These data imply that the Ras pathway and pathways mediated by sex-linked gene products might somehow interact to modulate an individual’s eventual phenotype. However, to date, there is little information available on whether gender is a significant factor in the manifestation of attentional problems and ADHD in NF1 and this is an avenue that may be useful to explore in future work. Interestingly, individuals presenting with both NF1 and Turner syndrome have been reported, highlighting the clinical similarities between the two conditions and the possibility of a partially overlapping molecular pathology [99].

NF1 deficient patients and/or rodents show subtly altered CNS neuronal morphology including shorter neurite lengths, smaller growth cone areas and attenuated survival [87], altered dendritic spine number [100] and increased perfusion and density of microglia in the amygdala [101]; these measures have yet to be formally assessed in TS and associated models. Unlike in NF1-deficient subjects, to date, there is little evidence for altered neurochemistry in TS patients or the 39,XO mouse [26, 102]. However, this dearth of information is likely to be due to a lack of systematic investigations in this area rather than a definite absence of karyotype-dependent effects. Small, but significant, changes in GABAergic receptor gene expression have been reported in the 39,XO whole mouse brain [103], and so, potentially, GABAergic system dysfunction may partially underlie attention deficits in both NF1 and TS.

Further clues as to the shared molecular pathology underlying ADHD phenotypes between TS and NF1 may come from a comparison of gene expression in the two relevant mouse models (39,XO mouse and NF1 heterozygous knockout mouse), or cell lines from these mutants (see above) or patients. A comparison of whole brain gene expression in 40,XX and 39,XO mice revealed only a very small number of significantly differentially expressed genes [104] including the X-inactivation escapees *Eif2s3x*, *Utx* and *Ddx3x*, none of which have previously been implicated in ADHD. To reiterate, the relevance of the 39,XO mouse model for understanding the molecular neuropathology of TS is debatable given the lack of X-escapees in mice relative to humans [27]. Gene expression studies in heterozygous NF1 knockout mice have focussed on the hippocampus (largely because of its key role in learning, memory and spatial processes). These analyses have highlighted effects of the mutation on the expression of genes encoding plasticity-related synaptic proteins [105] and kinesins [90] but do not identify *Eif2s3x*, *Utx* or *Ddx3x* as differentially expressed genes. Hence, we may have failed to detect true brain gene expression commonalities between TS and NF1 models (perhaps due to using whole brain or hippocampal tissue respectively), or there truly is little overlap on this measure between the two models. Addressing which of these two possibilities is correct could be done through more relevant comparisons e.g. comparing gene expression in matched rodent brain tissues, or in induced pluripotent stem cell (iPSC)-derived neuronal cells from individuals affected by one or other of the two conditions.

Given the current paucity of data regarding the neuroanatomy of NS patients, work in model systems will be fundamental to understanding the neural correlates of the cognitive and attentional deficits seen in this group. Data from genetically engineered mouse models and hippocampal slices harbouring equivalent *Ptpn11* mutations to those seen in cases of NS, have revealed abnormal myelination patterns [94], increased baseline excitatory synaptic function and deficits in long-term potentiation (LTP) and spatial learning [95]; the LTP and cognitive deficits could be normalised by administration of lovastatin, which reduces activation of the GTPase Ras-extracellular signal-related kinase (Erk) pathway in the brain. Currently, neither attentional function, nor the neurochemical basis of the cognitive deficits, have been assessed in these promising models, so it remains to be seen whether the genetic mutation recapitulates the ADHD phenotypes commonly observed in NS, and, if so, whether targeted therapeutic options may alleviate this. A priori, one might expect to see some similar neural phenotypes in the *Ptpn11* models and the *NF1* knockout model and it will be interesting to see the extent to which these overlap. In contrast, one might also expect to find differing neural phenotypes in these models given the opposing effect of these mutations on neurotransmitter phenotypes. *Ptpn11* mutation increases basal excitatory synaptic transmission in hippocampal neurons and has a role in postsynaptic glutamatergic neurons through enhanced trafficking of AMPA receptors and effect on LTP dependent learning and memory [95]. *NF1* mutation increases inhibitory synaptic transmission in hippocampal neurons, and has a role in presynaptic neurons through increased GABA release [80, 81].

## Conclusions

Whilst we now know that ADHD rates are elevated in Turner syndrome and the RASopathies, we still have very little information on ADHD subtypes and co-morbidities in these populations, about how ADHD-related phenotypes vary with age in these conditions, and about their underlying psychological, neural and molecular substrates. Therefore, more detailed clinical, neuropsychological and neuroanatomical phenotyping across cases and appropriate neurotypical controls across development is required; there also should be an emphasis on measures of brain function (e.g. EEG, fMRI) particularly during tasks taxing ADHD-related constructs such as alerting, orienting and executive control [106], with a view to more accurately specifying the neural systems that are particularly perturbed in TS and RASopathies, and identifying the extent to which the same systems are perturbed in these conditions and idiopathic ADHD. Studies into rare conditions are necessarily hampered by low power; hence, it will be important, perhaps through collaboration, to assess large patient samples from diverse geographical regions, in order to account for factors such as age and medication history, and to stratify samples on the basis of genotype to enable informative and robust genotype-phenotype correlations to be drawn. Such studies will not only improve our knowledge of the specific pathophysiological mechanisms underlying particular phenotypes and their amenability to treatment, but will be of importance for genetic counselling.

The brains of patients with TS or a RASopathy cannot be studied intimately in vivo. Hence, useful animal and cellular models for these conditions have been, and will continue to be, critically important for understanding the basis of associated cognitive deficits. There are now a number of excellent behavioural assays available for mice that tax processes of relevance to ADHD, many of which are conceptually and functionally analogous to human tasks for maximal translational value [41, 107–109]. Hence, the parallel assessment of brain structure, and function at various levels (e.g. electrophysiological, neurochemical via in vivo microdialysis, or imaging via *c-fos* immunohistochemistry or fMRI) during performance of such tasks, in TS and RASopathy models will enable high resolution of the aberrant neural circuitry underlying any cognitive phenotype; such predictions may then be tested experimentally through techniques such as optogenetics or DREADDs. Further work in iPSCs originating from TS or RASopathy patient samples, or their neuronal derivatives, will also provide clues as to molecular processes that may underlie ADHD-related phenotypes, and that could be followed up further in animal models and, ultimately, patients.

Studies explicitly designed to look for commonalities and dissociations in abnormal brain structure or molecular dysfunction across conditions and models with ADHD-like phenotypes may also be undertaken to identify and characterise pathways that may frequently be disturbed in ADHD. This approach has recently been taken with regard to neuroimaging phenotypes in 26 mouse models recapitulating features of autism spectrum conditions (including the 39,XO mouse) [110], and could feasibly be undertaken with existing rodent models for TS, NF1 and NS.

New insights into the molecular mechanisms predisposing to ADHD in TS may soon come from genome-wide genetic screens in idiopathic ADHD. To date, genome-wide association studies, copy number variant studies and exome/whole genome sequencing studies in ADHD have been limited by low power, a particular concern where the aetiology of the disorder is thought to be multifactorial and due to a complex mix of common and rarer variants. Moreover, these studies have traditionally neglected the X chromosome for a variety of reasons. With more ADHD samples now available for genotyping, a growing recognition that the X chromosome is influential in neurocognitive phenotypes, and better statistical tools for dealing with X-linked data, X-linked genes that are robustly implicated in idiopathic ADHD (and therefore that may play a key role in ADHD risk within TS) may soon be identified. Large-scale genome-wide studies in well-characterised ADHD samples may also potentially implicate genes involved in the Ras signalling cascade, including those involved in NF1 and NS; through interrogation of the clinical phenotypes associated with each variant, a clearer picture of how the various gene mutations may affect cognition and behaviour could be drawn.

## Abbreviations

ADHD: Attention deficit hyperactivity disorder
CFC: Cardiofaciocutaneous syndrome
CS: Costello syndrome
NF1: Neurofibromatosis type 1
NS: Noonan syndrome
TS: Turner syndrome
